# The use and ethics of dental photography and social media at an oral healthcare training centre in South Africa

**DOI:** 10.4102/hsag.v29i0.2590

**Published:** 2024-07-31

**Authors:** Faheema Kimmie-Dhansay, Nicoline Potgieter, Nanayaa Mprah, Lindeka Msane, Shaza Mowzer, Azraa Mowzer, Nosipho Mthupha, Tamiya Safodien, Mirriam Sindani, Jade Smith, Olwethu Solombela, Zahraa Suliman

**Affiliations:** 1 Department of Community Dentistry, Faculty of Dentistry, University of the Western Cape, Cape Town, South Africa; 2 Department of Pediatric Dentistry, Faculty of Dentistry, University of the Western Cape, Cape Town, South Africa

**Keywords:** dental photography, ethics, students, higher education, Protection of Personal Information, informed consent, social media

## Abstract

**Background:**

The ethics surrounding the use and sharing of photographs on social media has come under the spotlight as the *Protection of Personal Information Act* (POPI Act) has come into play.

**Aim:**

The aim is to determine the use, ethical practice and sharing of dental photography on social media among qualified and undergraduate oral health practitioners at a dental school in South Africa.

**Methods:**

A cross-sectional study design was used on staff and students at the University of the Western Cape’s Dental Faculty in 2022. Chi-squared and Fisher’s exact tests were used to determine associations between the different graduation statuses and various demographic factors.

**Results:**

From the 80 undergraduate students and 46 qualified oral healthcare practitioners, the majority were aware that photography could be used in dentistry, and 87.3% (*n* = 110) took photographs of the dental treatments performed on their patients. Only 60.3% of the participants attended an ethical course that addressed issues with social media and digital photography. Almost 80% (*n* = 100) of the participants did not feel that they needed to mention all the social media platforms that they would use with their patients’ photographs before sharing.

**Conclusion:**

Dental photography is being used and sometimes shared on social media platforms by some students and staff at university level. Not all participants have attended an ethical course on clinical photography. Dental training needs to include an ethical course on dental photography and the use of sharing photographs on social media.

**Contribution:**

Good ethical practice regarding clinical photographs in all undergraduate and postgraduate curriculums, to eliminate any uncertainty.

## Introduction

Dental photography usage has been on the increase with cameras being available on most mobile phones. With the change in online and hybrid education practices, there is an increased need for high-quality clinical photographs. Digital photography is frequently the most effective method of collecting and preserving evidence, forensic documentation, marketing, legal documentation, education, consultation and journal publications (Hannah et al. 2018). Dental photography in the future can also be used as a training tool (McDonnell & Newsome 2011) and in diagnostic accuracy tests (Jackson et al. 2019).

Regardless of the reason for taking clinical photographs, it is critical to receive written consent from the patient and maintain confidentiality before taking any photographs (Roguljić et al. 2022). Every human being has the right to provide their consent before their personal information is used. Similarly, depicting any individual requires their consent.

Sharing of photographs has become affiliated with social media. In January 2022, Google, YouTube, Facebook and Wikipedia were the four most visited websites in November 2021, documented as over 7.6 billion visitors (McLachlan 2023). Instagram had 3.1 billion visits in the same month.

The *Protection of Personal Information Act* (POPI Act) (2013) defines the required and minimum standards with regard to accessing and the ‘processing’ of any form of personal information belonging to another individual POPI Act (Western Cape Government 2020). The POPI Act’s ultimate mission is to protect the processing of personal information by both public and private entities. The act is significant for all South Africans because it protects the distribution and prevents the misuse of personal information by individuals and corporations both domestically and internationally (Buys 2017).

Ethics forms the foundation of decision making in the medical practice and includes the principles of autonomy, non-maleficence, beneficence and justice (Simplício 2019). Each person has the right to decide on treatment, interventions and the sharing of information pertaining to themselves. Informed consent therefore also refers to respecting patient’s choices and treating patients with dignity. There is a difference between giving consent to dental treatment and giving consent to the use of images (Sykes et al. 2017a). Consent for the use of dental images involves the clinician providing adequate information on how the dental images will be shared. The consent should be obtained from the person being photographed, or in the case of a minor, the caregiver or parent should give informed consent (Abouzeid et al. 2020; Cunniff & Mostert 2012). Assent should also be obtained from the minor. A list of all the possible media publications should be mentioned when informed consent is taken. The patient should be made aware that once an image is posted publicly, the image will not be able to be removed. Patients should also be made aware that they can refuse the use of their images for publication (Cunniff & Mostert 2012).

The aim is to determine the ethical practice and sharing of dental photography on social media among qualified and undergraduate oral health practitioners at a dental school in South Africa.

## Research method and design

A cross-sectional study design was used. The sample included 656 participants of which 93 were staff members (83 dentists and 10 oral hygienists) and 255 registered postgraduate students (dentists and oral hygienists). The study included registered dentistry and oral hygiene students of the University of the Western Cape’s Faculty of Dentistry. A database of all registered Bachelor of Oral Hygiene (BOH) II and III as well as Bachelor of Dental Surgery (BDS) III, IV and V students was used. A convenience sampling method was used for 308 students, 34 students from BOH II; 28 from BOH III; 80 from BDS III; 79 from BDS IV and 89 from BDS V.

The sample size was calculated according to the formula:


$$m=\frac{\left(\frac{\text{Z}\alpha }{2}\right)2\text{p}(1-\text{p})*\text{D}}{d^{2}}=\frac{{\left(1.96\right)}^{2}*0.787.(0.213)}{0.05^{2}}=257.59$$ [Eqn 1]

$$n=\frac{m}{1+\frac{m-1}{N}}=\frac{257.59}{1+\frac{257.59-1}{656}}=185$$ [Eqn 2]

where *n* = sample size, *Z* = statistical level of confidence, *p* = expected proportion and *d* = precision. If *Z* = 1.96 (95% confidence) and using a prevalence of 78.7% (Alharbi 2021), *p* = 0.787 and *d* = 0.05, *D* = design effect = 1, *N* = 656, *n* is approximately 185. A study sample of 185 participants was expected.

### Inclusion criteria

Registered postgraduate dental, postgraduate oral hygiene students, full-time or part-time staff members, all undergraduate dental and undergraduate oral hygiene students at the UWC Faculty of Dentistry, who provided electronic consent, were included in this study.

### Exclusion criteria

Any of the eligible participants who did not give informed consent and who were not students or a staff member at UWC and those who were unable to give consent were excluded were excluded from this study.

### Data collection and analysis

Once ethical approval was obtained, data were collected, between April and August 2022, through the use of REDcap© as an online data-capturing platform. The individuals who were eligible to participate in the study were emailed by the UWC administrator with a request to participate in the study. To ensure the validity and reliability of the questionnaire, the questionnaire published by Hannah et al. (2018) was adapted for this study. The questionnaire consists of 28 close-ended questions and includes the demographic information of the respondents and various bivariate and specific multiple-choice questions to gather information about their use of dental photography.

The questionnaire was used to obtain personal information about each respondent, such as the person’s gender, age, study programme they were registered for or if they are a full-time or sessional staff member at UWC was collected. The use and ethics of dental photography, each had 14 and 11 close-ended questions, respectively.

The information sheet and questionnaire were in English, as all undergraduate, postgraduate and staff are conducting their teaching and training activities in English. All participants were therefore able to understand the information sheet and answer the questionnaire.

After capturing the data on REDcap©, the information was downloaded as an Excel spreadsheet, anonymised and imported into the STATA software for statistical analysis (StataCorp. 2019. Stata Statistical Software: Release 17. College Station. TX: StataCorp LLC.). Descriptive Statistics (i.e. frequencies, percentages, means, standard deviations) and first-order analysis (i.e. chi-square tests and Fisher’s exact test) were performed. All tests were deemed statistically significant at *p* < 0.05.

### Ethical considerations

All candidates were informed that participation in this study was entirely voluntary, that informed consent was mandatory and that they had the freedom to withdraw from the study at any time during the research study without penalty. There were no risks in participating in this study. Permission from the Dean of the dental faculty was granted to involve students and staff in this project. Ethical clearance was obtained from the Biomedical Research Council at the University, the ethics approval number is BM22/4/8.

## Results

There were 126 participants in this study, representing a response rate of 19.2%. There were 80 undergraduate students and 46 qualified oral healthcare practitioners. The median age of the participants was 23 [IQR: 21–32]; the oldest participants were 65 and the youngest was 19. The majority of the participants were second-year undergraduate BDS students (55.5%, *n* = 70), which comprised 70% of the undergraduate students (Table 1). Although there were more females (69.8%, *n* = 88) than males (30.2%, *n* = 38), there was no association between sex and qualification type, *p* = 0.247.

**TABLE 1:** Characteristics of the participants.

Characteristics	*n*	%
**Sex**		
Female	88	69.8
Male	38	30.2
**Type of undergraduate student**		
Undergraduate BOH student	10	7.9
Undergraduate BDS student	70	55.6
**Type of qualified oral healthcare practitioner**		
Full-time staff member	12	9.5
Part-time staff member	11	8.7
Postgraduate BDS student	23	18.3

BOH, bachelor of oral hygiene; BDS, bachelor of dental surgery.

### Use of dental photography

**FIGURE 1:**
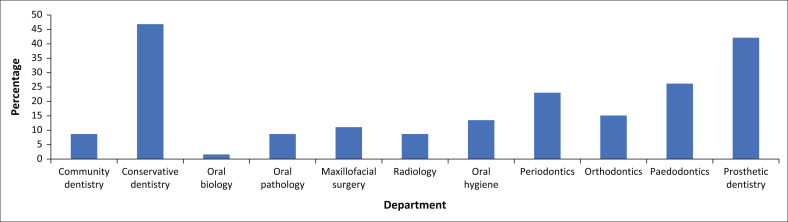
Departments in which dental photographs were taken in.

The majority of the participants were aware that photography could be used in dentistry (Table 2) and 87.3% (*n* = 110) took photographs of their dental treatments performed on their patients. Neither of the above was statistically significantly different by qualification status (*p* = 0.079). The most common tool, 14.3% (*n* = 18), used to take photographs was a phone camera (Table 2). Participants could choose as many reasons as they wished to display their reason for taking dental photographs and most were for clinical assessment (64.3%, *n* = 81) to monitor treatment (49.2%, *n* = 62) and research purposes (48.4%, *n* = 61). Most of the photographs were taken in the Department of Conservative Dentistry (46.8%, *n* = 59) followed closely by the Department of Prosthetics (42.1%, *n* = 53) (Figure 1). The Department of Radiology and Pathology was reported to have the least number of photographs taken (Table 2). Almost all participants believed that clinical photographs were essential for teaching and training (Table 2). Almost 10% of the participants completed a dental photography course (Table 2), while 60.3% (*n* = 76) attended an ethics course that addressed issues with social media and digital photography (Table 2).

**TABLE 2:** Use of dental photographs.

Question	Total		Undergraduate		Qualified		*p*
	*n*	%	*n*	%	*n*	%	
**Are you aware that photography can be used in dentistry?**							1.000
No	1	0.8	1	100.0	0	0.0	
Yes	125	99.2	79	63.2	46	36.8	
**Do you take photographs of your dental treatment?**							0.079
No	16	12.7	7	43.8	9	56.2	
Yes	110	87.3	73	66.4	37	33.6	
**Do you believe that clinical photographs are essential for teaching and training?**							0.451
No	1	0.8	1	100.0	0	0.0	
Yes	124	99.2	79	63.7	45	36.3	
**Have you attended a dental photography course?**							0.099
No	114	90.5	75	65.8	39	34.2	
Yes	12	9.5	5	41.7	7	58.3	
**Have you attended an ethics course that addresses issues with social media and digital photography?**							0.218
No	50	39.7	35	70.0	15	30.0	
Yes	76	60.3	45	59.2	31	40.8	
**Which tool do you use to take photographs?**							-
Phone camera	102	81.0	73	71.6	29	28.4	
Digital camera	18	14.3	6	33.3	12	66.7	
DSLR	12	9.5	1	8.3	11	91.7	
iPad	1	0.8	1	100.0	0	0.0	
Other_camera	10	7.9	4	40.0	6	60.0	
**Reasons for taking photographs?**							-
Patient education	56	44.4	30	53.6	26	46.4	
Treatment plan	54	42.9	30	55.6	24	44.4	
Monitor treatment	62	49.2	35	56.5	27	43.5	
Medico-legal	20	15.9	3	15.0	17	85.0	
Research	61	48.4	38	62.3	23	37.7	
Clinical assessment	81	64.3	57	70.4	24	29.6	
Lectures	26	20.6	7 26.9		19	73.1	
**In which clinic do you take photographs in?**							-
CommDent	11	8.7	5	45.4	6	54.6	
Cons	59	46.8	48	81.4	11	18.6	
Biology	2	1.6	0	0.0	2	100.0	
Path	11	8.7	7	63.6	4	36.4	
Maxfac	14	11.1	11	78.6	3	21.4	
Radio	11	8.7	9	81.8	2	18.2	
OH	17	13.5	13	76.5	4	23.5	
Perio	29	23.0	21	72.4	8	27.6	
Ortho	19	15.1	10	52.6	9	47.4	
Paedo	33	26.2	16	48.5	17	51.5	
Prosthetic	53	42.1	51	96.2	2	3.78	

DSLR, digital single-lens reflex; OH, oral hygiene.

### Use of social media in dentistry

Almost 95% of the sample had a social media account, 94.4% (*n* = 119) (Table 3), and there was no statistically significant difference in qualification status and the creation of a social media account (*p* = 0.72). Some participants had multiple social media accounts, with WhatsApp being the most common platform and Snapchat being the least common social media platform (27.8%, *n* = 35) (Table 3). Even though most participants owned a social media account, not that many shared their photos on that platform. The most common platforms used to share dental photographs were WhatsApp (46.8%, *n* = 59) and Instagram (31.8%, *n* = 40).

**TABLE 3:** Cell phone ownership and social media characteristics of participants.

Question	*n*	%	Undergraduate		Qualified	
			*n*	%	*n*	%
**Do you have a social media account?**						
No	7	5.6	4	57.1	3	42.9
Yes	119	94.4	76	63.9	43	36.1
**Do you have a smartphone?**						
No	1	0.8	1	100.0	0	0.0
Yes	125	99.2	79	63.2	46	36.8
**Do you feel disadvantaged by not having a camera on your phone?**						
No	93	74.4	58	62.4	35	37.6
Yes	32	25.6	21	65.6	11	34.4
**Do you have a professional camera with a ring flash?**						
No	99	79.2	69	69.7	30	30.3
Yes	26	20.8	10	38.5	16	61.5
**Do you feel disadvantaged by not having a camera and ring flash?**						
No	75	75.8	56	74.7	19	25.3
Yes	24	24.2	13	54.2	11	45.8

Most (99.2%, *n* = 125) of the participants had a smartphone. In addition, most of the participants (79.2%, *n* = 99) did not have a professional camera with a ring flash, and the majority of those participants did not feel disadvantaged by it (75.8%, *n* = 75).

### Ethical characteristics

**FIGURE 2:**
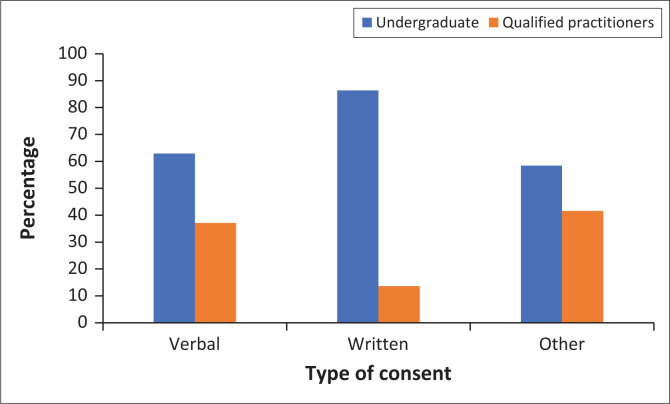
Type of consent requested by oral healthcare practitioner.

Almost 98% (*n* = 122) of participants were aware that they needed informed consent when taking dental photographs, and there was no statistically significant difference between graduation status and the awareness of needing this informed consent (*p* = 0.5545) (Table 4). In addition, the majority knew that they needed informed consent from patients when using personal patient photos on social media, and there was no statistically significant difference between graduation status (*p* = 1) (Table 4). The majority (80.8%, *n* = 101) felt that written consent was enough, while 17.6% (*n* = 22) felt that verbal consent was enough (Figure 2). In addition, there was a statistically significant association between the type of consent needed (verbal vs. written) and graduation status (*p* = 0.045*).

**TABLE 4:** Ethical characteristics of the study sample.

Question	*n*	%	Undergraduate		Qualified		*p*
			*n*	%	*n*	%	
**Are you aware that you need informed consent for dental photography?**							0.5545
No	3	2.4	1	33.3	2	66.6	
Yes	122	97.6	78	63.9	44	36.1	
**Do you need informed consent from patients when using their photographs on SM?**							1.000
No	1	0.8	1	100.0	0	0.0	
Yes	124	99.2	78	62.9	46	37.1	
**What type of consent is needed?**							0.045*
Verbal	22	17.6	19	86.4	3	13.6	
Written	101	80.8	59	58.4	42	41.6	
Other	2	1.6	1	50.0	1	50.0	
**Do you maintain patient confidentiality when presenting patient photographs?**							0.434
Yes	121	96.0	76	62.8	45	37.2	
No	5	4.0	4	80.0	1	20.0	
**Do you think that your method of maintaining confidentiality is sufficient?**							0.254
No	14	11.1	11	78.6	3	21.4	
Yes	112	88.9	69	61.6	43	38.4	
**Have you heard of the POPI Act?**							0.457
No	8	6.4	4	50.0	4	50.0	
Yes	117	93.6	76	64.9	41	35.1	
**When obtaining consent, do you have to mention all forms of SM that you are going to use?**							0.822
No	26	20.6	17	65.4	9	34.6	
Yes	100	79.4	63	63.0	37	37.0	
**Do you state that the patient consented to posting their picture on SM?**							0.856
No	29	23.0	18	62.1	11	37.9	
Yes	97	77.0	62	63.9	35	36.1	
**Have you shared a patient photograph/X-ray on a SM platform?**							0.401
No	65	51.6	39	60.0	26	40.0	
Yes	61	48.4	41	67.2	20	32.8	
**Which platform did you use to share the photograph/X-ray?**							**-**
TikTok	3	2.38	3	100.0	0	0.0	
Instagram	40	31.8	33	82.5	7	17.5	
Twitter	2	1.6	2	100.0	0	0.0	
Facebook	8	6.4	2	25.0	6	75.0	
WhatsApp	59	46.8	40	67.8	19	32.2	
Snapchat	3	2.4	3	100.0	0	0.0	
Other	40	31.8	22	55.0	18	45.0	

POPI, Protection of Personal Information; SM, social media.

* , *p*-value is < 0.05.

**FIGURE 3:**
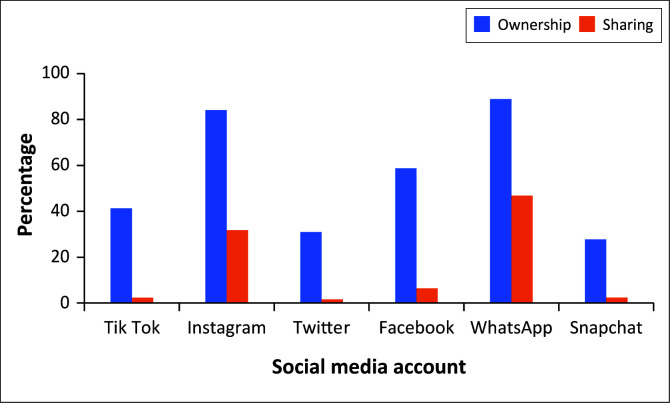
Social media accounts held by participants.

More than 50% of the participants have not shared a photograph of their patients on social media (51.6%, *n* = 65). The majority of the participants (96.0%, *n* = 121) felt that it was necessary to maintain patient confidentiality when sharing patient photographs, and 88.9% (*n* = 112) felt that they did a good job in maintaining confidentiality. Covering their patients’ eyes, masking unique features, using photoshop and removing names and folder numbers were some ways used to de-identify patients (Table 4). In addition, the undergraduate students were more likely to cover up their patients’ identities compared to the graduated oral healthcare practitioners (Figure 3). Almost 80% (*n* = 100) of the participants did not feel that they needed to mention all the social media platforms that they would use with their patients’ photographs before sharing. A total of 23.0% (*n* = 29) of participants admitted to sharing photographs on social media without obtaining consent from the patient.

## Discussion

The results obtained from this study, conducted among the dental students and staff members, indicate that 87.3% of the participants take photographs. The possible reasons for participants not taking clinical photographs could be a lack of photography skills, not having a smartphone, not perceiving it as important, not having expensive equipment, cross-contamination or insufficient time (Yousuf, Jan & Sidiq 2020).

The most popular device among the participants, in this study, was a phone camera as it is at their disposal at all times. Our study’s findings, about the majority of phone camera usage, were corroborated by the findings from Abouzeid et al. (2020) and Alghulikah (2022). What was interesting to explore was how clinical assessment, research, monitoring treatment, patient education and treatment planning were the main reasons why participants took dental photographs. This was also seen in Abouzeid et al. (2020) and Jamal et al. (2022), who reported that patient education was the main reason why dental photographs were taken. While Alghulikah (2022) saw documentation, treatment planning and discussions with patients, as the major reasons for taking dental photographs. Meanwhile, Kashyap et al. (2014) used dental photography for different reasons: to avoid post-dental problems, educate the patient about their own treated cases, as a helping hand to solve dento-legal cases and for maintaining better patient–doctor relations.

The most common departments where dental photographs were taken were in Conservative Dentistry and Prosthetic Dentistry. Both these departments are focused on achieving a functional and aesthetic outcome. Photographs are used for treatment planning and drastic changes or improvements are often visible and documented, which may explain this finding. Living in a digitally inclined world it was not surprising to see that 94.4% of students have some form of a digital footprint (social media) but what was concerning with this finding was that only 60.3% have attended an ethical course that addresses issues with social media. The participants have demonstrated that attending a course on dental photography is not as important as attending a course on the ethics of dental photography. Moreover, dental photography courses in South Africa are very expensive.

According to Goodchild and Donaldson (2023), point-and-shoot, Digital single-lens reflex (DSLR), mirrorless interchangeable lens and even smartphone cameras are all examples of digital cameras that become outdated nearly as they reach the market. Recent evidence indicates that the availability and calibre of smartphone cameras are causing a decline in the market for point-and-shoot, DSLR and mirrorless cameras. Although taking dental photographs with a smartphone might be possible, there are potential macro lens restrictions, poor flash alternatives and patient confidentiality requirements that make it less than ideal. However, in some circumstances, using a smartphone can deliver decent photographs. Monitoring the digital camera’s infection control is a challenge during the production of high-quality dental photographs. Cross-contamination is normally not an issue while using an intraoral camera because there are disposable barrier sleeves available, but it might be difficult to avoid when using a smartphone, point-and-shoot, DSLR or mirrorless camera (Goodchild & Donaldson 2023).

With WhatsApp and Instagram being the most common application, having easy access to social media via your mobile phone can result in mindless and unethical practices in the sharing of patients’ data. Care should be taken to make sure that patients’ identities are masked at all times. The majority of the participants knew they needed informed consent to take dental photographs and to obtain either verbal or written consent before sharing photographs.

Obtaining informed consent for clinical photographs requires providing an adequate description to the patient about the photos’ intended use and purpose, the target audience, the extent of patients’ information that will be disclosed and the potential domains where they might appear. All patients’ photographs must follow the same approval procedures, confidentiality, assurances and security measures as with any other medical record, regardless of whether or not they will be seen by others or contain identifiable characteristics (Sykes et al. 2017b). The *Protection of Personal Information Act* (POPI Act)’s principal goal is to safeguard the handling of personal data by both governmental and commercial entities (Buys 2017). In this study, almost everyone was aware of the POPI Act.

Patients’ confidentiality in photographs should be maintained unless it is extremely necessary to show the complete picture. There are a few ways patient confidentiality can be achieved namely, using photo editing software, concealing or blurring out their eyes and masking features that are unique to or that characterise the patient (Abouzeid et al. 2020). Other features such as jewellery, scars and tattoos should be kept in mind as these are identifiable features (Abouzeid et al. 2020).

There was no difference in the use, ethics or social media sharing of photographs between undergraduate and qualified oral healthcare practitioners

### Recommendations and limitations

A limitation of this study was the poor response rate and the resulting small sample size. There are various studies performed on staff and students throughout the year, and the authors presume that the participants could have suffered from participation fatigue. Further research with a larger sample size is recommended to also include general dental practitioners or healthcare workers in the private sector or not affiliated to an academic institution, as well as students and staff members at other academic institutions.

## Conclusion

The result of the study confirms that dental photography is being used and sometimes shared on social media platforms by some students and staff at the university level. Photographs were mostly taken for teaching and treatment planning. Most participants agreed that informed consent was required for photographs. Not all participants have attended an ethical course including dental photography. Although most participants were aware of the POPI Act the need to obtain informed consent for the taking and sharing of dental photography, it was not always practiced correctly. Undergraduate and post-graduate training needs to include an ethical course on dental photography and the sharing of photographs on social media.
